# Thermophilic and alkaliphilic *Actinobacteria*: biology and potential applications

**DOI:** 10.3389/fmicb.2015.01014

**Published:** 2015-09-25

**Authors:** L. Shivlata, Tulasi Satyanarayana

**Affiliations:** Department of Microbiology, University of DelhiNew Delhi, India

**Keywords:** *Actinobacteria*, thermophiles, alkaliphiles, polyextremophiles, bioactive compounds, enzymes

## Abstract

Microbes belonging to the phylum *Actinobacteria* are prolific sources of antibiotics, clinically useful bioactive compounds and industrially important enzymes. The focus of the current review is on the diversity and potential applications of thermophilic and alkaliphilic actinobacteria, which are highly diverse in their taxonomy and morphology with a variety of adaptations for surviving and thriving in hostile environments. The specific metabolic pathways in these actinobacteria are activated for elaborating pharmaceutically, agriculturally, and biotechnologically relevant biomolecules/bioactive compounds, which find multifarious applications.

## Introduction

The phylum *Actinobacteria* is one of the most dominant phyla in the bacteria domain (Ventura et al., 2007), that comprises a heterogeneous Gram-positive and Gram-variable genera. The phylum also includes a few Gram-negative species such as *Thermoleophilum* sp. (Zarilla and Perry, 1986), *Gardenerella vaginalis* (Gardner and Dukes, 1955), *Saccharomonospora viridis* P101^T^ (Pati et al., 2009), *Ferrimicrobium acidiphilum*, and *Ferrithrix thermotolerans* (Johnson et al., 2009). Actinobacteria are either aerobes or anaerobes, motile or non-motile, and spore-/non-spore forming bacteria with a high G+C content (>55 mol%; Ensign, 1992). The genome size of actinobacteria ranges from 0.93 Mb (*Tropheryma whipplei*; Bentley et al., 2003) to 12.7 Mb (*Streptomyces rapamycinicus*; Baranasic et al., 2013), that exists either as a circular or linear form. Actinobacteria occur in diverse ecological niches such as terrestrial and aquatic ecosystems (fresh and marine waters), characterized by a complex life cycle that includes their existence either as dormant spores or actively growing hyphae. They are highly diverse in their morphology ranging from coccoid (e.g., *Micrococcus*) and rod-coccoid (e.g., *Arthrobacter*), fragmenting hyphal forms (e.g., *Nocardia*) to branched mycelium (e.g., *Streptomyces*; Barakate et al., 2002). Reproduction in actinobacteria occurs either by vegetative mode via fragmentation of mycelia or by asexual mode (spore or conidia formation). They produce either a single spore (monosporic) or a pair of spores (bisporic), or many spores (oligosporic) on aerial or substrate mycelium. The oligosporic actinobacteria show distinct patterns of spore arrangement (hooked, straight, or wavy) on the mycelium, depending on the taxa.

Actinobacteria represent one of the most primitive lineages among prokaryotes (Koch, 2003) which are believed to have evolved about 2.7 billion years ago (Battistuzzi and Hedges, 2009). Antibiotic production by actinobacteria is considered to be a key driving factor in the evolution of prokaryotes that led to the diversification of archaea and Gram-negative bacteria (diderm) from Gram-positive bacteria (monoderm; Gupta, 2011). Actinobacteria form a distinct branch on the 16S rRNA gene tree (Zhi et al., 2009), and are distinguished from other bacterial taxa on the basis of their distinct gene arrangement patterns (Kunisawa, 2007) and conserved indels present in both the 23S rRNA and proteins (e.g., cytochrome C oxidase subunit I, CTP synthetase, and glutamyl-tRNA synthetase; Gao and Gupta, 2005). Their classification has been revised many times in the past. According to the recent system of classification, these are placed under Phylum XXVI, *Actinobacteria* in the Domain II (Bacteria) in Bergey's Manual of Systematic Bacteriology, volume 5. This phylum contains a large array of chemotaxonomically, morphologically and physiologically distinct genera, grouped into six major classes (*Actinobacteria, Acidimicrobiia, Coriobacteria, Nitriliruptoria, Rubrobacteria*, and *Thermoleophilia*; Goodfellow et al., 2012).

Actinobacteria are an ecologically significant group, which play a vital role in several biological processes such as biogeochemical cycles, bioremediation (Chen et al., 2015), bioweathering (Cockell et al., 2013), and plant growth promotion (Palaniyandi et al., 2013). They not only produce a large array of pharmaceutically important bioactive compounds (antibiotics, antitumor agents, anti-inflammatory compounds, and enzyme inhibitors) but also an enormous number of industrially and clinically important enzymes. Since the discovery of streptomycin (first discovered antituberculosis drug from actinobacteria), the drug discovery and development programmes have inclined toward the antimicrobial agents than chemical compounds. Subsequently, a large number of actinobacterial species have been searched for the discovery of clinically valuable compounds. The phylum *Actinobacteria* contains several genera encompassing antibiotic producing species. The genus *Streptomyces* is a prominent source of secondary metabolites, especially antibiotics. *Streptomyces* species are known to produce more than 50% of the total known microbial antibiotics (≥10,000). Despite the availability of enormous number of clinical drugs, many pharmaceutical companies and research laboratories are engaged in the search for new therapeutic drugs in order to combat the microbial pathogens. Multidrug resistant pathogenic strains are constantly emerging, which cause severe disease outbreaks in several countries. In order to find novel bioactive compounds of pharmacological and industrial relevance, actinobacteria have been isolated from exotic and unexplored locations such as desert (Kurapova et al., 2012), marine (Manivasagan et al., 2013), and wetland (Yu et al., 2015) areas. On the premise that the extremophilic actinobacteria could be a source of new valuable metabolites (Bull, 2010) with gene clusters for the synthesis of novel biomolecules, attempts are being made to isolate actinobacteria from extreme environments.

## Extremophilic/extremotolerant actinobacteria

Actionobacteria are known to occur not only in normal environments, but also in extreme environments, which are characterized by acidic/alkaline pH, low or high temperatures, salinity, high radiation, low levels of available moisture, and nutrients (Zenova et al., 2011). The diverse physiology and metabolic flexibility of extremophilic/extremotolerant actinobacteria enable them to survive under hostile and unfavorable conditions. The high abundance of actinobacterial species was recorded in all extreme environments (Bull, 2010) which had broken the traditional paradigm of restricted predominance of actinobacteria in soil and fresh water habitats. Enormous data has been reported on actinobacteria isolated from normal environments (neutral pH and temperature ranging 20–40°C). Only a few investigations have been carried out to understand the diversity of actinobacteria in the extreme environments, their ecological role and adaptation. Polyextremophiles and polyextremotolerant actinobacterial species also exist in environments with two or more extreme conditions. Polyextremophiles can adapt to environments with multiple stresses (Gupta et al., 2014), which include alkalithermophilic, thermoacidophilic, thermophilic radiotolerant, haloalkaliphilic, and thermoalkalitolerant actinobacteria. Their incidence has been documented in distinct extremes of geographical locations such as the Arctic (Augustine et al., 2012) and Antarctic (Gousterova et al., 2014) regions, oceans (Raut et al., 2013), hot springs (Chitte and Dey, 2002), and deserts (Kurapova et al., 2012).

The extremophilic actinobacteria exhibit several adaptive strategies such as antibiosis, switching between different metabolic modes (i.e., autotrophy, heterotrophy, and saprobes) and production of specific enzymes to survive under unfavorable environmental conditions (high temperature, alkaline, and saline). The thermotolerance is attributed to the presence of high electrostatic and hydrophobic interactions and disulfide bonds in the proteins of thermophiles (Ladenstein and Ren, 2006). They have certain special proteins known as chaperones which aid in refolding the partially denatured proteins (Singh et al., 2010). Several other proteins are also synthesized that bind to DNA and prevent their denaturation at elevated temperatures. Some actinobacteria have acquired multiple adaptive mechanisms to survive in environments with two or more stresses. A thermophilic *Streptomyces* sp., isolated from desolated place, produced enzymes of the autotrophic metabolic pathway such as carbon monoxide dehydrogenase (CODH; Gadkari et al., 1990). The enzyme CODH facilitates the microbial growth in nutrient deprived condition by oxidizing the available inorganic compound such as carbon monoxide into CO_2_ which is further fixed by RuBisCO enzyme into microbial biomass through Calvin–Benson cycle (King and Weber, 2007). The thermophilic chemolithoautotroph, *Acidithiomicrobium* sp., isolated from geothermal environment, utilizes sulfur as an energy source (Norris et al., 2011). The antibiosis is another principal strategy through which actinobacteria sustain by killing other microbial flora under nutrient limited conditions. Acidophiles and alkaliphiles have acquired proton pumps to regulate H^+^ concentrations inside and outside the cell for maintaining physiological pH inside (Kumar et al., 2011). Alkaliphiles contain the negatively charged cell wall polymers which stabilize the cell membrane by reducing the charge density at the cell surface (Wiegel and Kevbrin, 2004). The adaptive strategy of haloalkaliphiles includes additional tolerances to the salt environment by synthesizing and accumulating high amount of compatible solutes (Roberts, 2005) that prevent desiccation through osmoregulation. They also have Na^+^/H^+^ antiporter to exclude excessive salt content from inside of the cell.

Actinobacteria are also known to show tolerance to extremely harmful radiations such as gamma and UV rays, and have been isolated from various radioactive sites. The three thermophilic *Rubrobacter* species such as *R. radiotolerans, R. xylanophilus* (Ferreira et al., 1999), and *R. taiwanensis* (Chen et al., 2004) have been reported to be radiotolerant. The resistance mechanism has not been adequately understood, but the complete whole genome analysis of *R. radiotolerans* RSPS-4 revealed the presence of genes encoding proteins involved in DNA repair system, oxidative stress response, and biosynthetic pathways of compatible sugars (trehalose and mannosylglycerate) which might be playing a role in mitigating the damage caused by radiations (Egas et al., 2014). In recent years, a few more alkalitolerant and radiotolerant actinobacterial species such as *Microbacterium maritypicum* (Williams et al., 2007), *Microbacterium radiodurans* GIMN 1.002T (Zhang et al., 2010), *Cellulosimicrobium cellulans* UVP1 (Gabani et al., 2012), *Kocuria* sp. ASB 107 (Asgarani et al., 2012), and *Kocuria rosea* strain MG2 (Gholami et al., 2015) have been documented. These two alkalitolerant *Kocuria* strains were isolated from Ab-e-Siah radioactive spring of Iran. The *Kocuria* sp. ASB 107 is a psychrotrophic strain which shows tolerance to ionizing radiation (upto 90% lethal doses) such as ultraviolet (256 nm-UV) and corona discharge. The *Kocuria rosea* strain MG2 was shown to endure the high dosage of harmful UV-C radiation. This actinobacterium can grow in a wide pH range (5–11 with optimum growth at pH 9.2) and salt concentration (0–15%). Gholami et al. (2015) performed the cell viability analysis on *Kocuria rosea* strain MG2 under multiple stresses. After 28 days of incubation under desiccation condition, the cells of *Kocuria* strain were found to be viable and showed high tolerance to the radiation and strong oxidant such as H_2_O_2_ (1–4%). The hydrogen peroxide is a well-known antimicrobial agent which damages biological membranes by generating hydroxyl radicals. They seem to exhibit both enzymatic (catalase and peroxidase) and non-enzymatic antioxidant defense systems (carotenoids) to diminish the effect of radiation or strong oxidants or other stresses (Gholami et al., 2015).

The resilience and adaptability of extremophilic/extremotolerant actinobacteria confer them a competitive advantage over other microbes. Besides helping them to survive under extreme conditions, the physiology and metabolic flexibility also trigger them to produce industrially valuable compounds (Singh et al., 2013). The production of biomolecules by extremophiles mitigates the risks of other microbial contaminations, besides providing thermostable, alkalistable, and halotolerant compounds. Enzymes produced by the extremophilic/extremotolerant actinobacteria are functional under extreme conditions, thus, making them suitable candidates for application in industrial processes, where harsh conditions/treatment methods are used. This review focuses on the physiology, phylogeny, ecological roles, and potential applications of thermophilic and alkaliphilic actinobacteria.

## Thermophilic and thermotolerant actinobacteria

Thermophilic actinobacteria thrive at relatively high temperatures ranging from 40 to 80°C (Tortora et al., 2007). They are widespread, commonly found in moldy hay (Corbaz et al., 1963), self-heating plant residues, cereal grains, sugar cane bagasse (Suihko et al., 2006), decaying vegetable materials, and compost heaps (Henssen and Schnepf, 1967). These are of two types: strictly thermophilic and moderately thermophilic actinobacteria. The former can grow in the temperature range between 37 and 65°C, but optimum proliferation takes place at 55–60°C. While moderately thermophilic actinobacteria thrive at 28–60°C and require 45–55°C for optimum growth (Jiang and Xu, 1993). Another group known as thermotolerant actinobacteria can survive at temperatures up to 50°C (Lengeler et al., 1999).

### Physiology

Thermophilic actinobacteria are strictly aerobes and obligate chemoorganotrophs in nature and thrive on decaying organic matter (dead animal and plant materials). There are certain thermophilic actinobacteria such as *Streptomyces thermoautotrophicus* (Gadkari et al., 1990) and *Acidithiomicrobium* sp. (Norris et al., 2011) which are obligate chemoautotrophs, growing solely on CO_2_+H_2_ and sulfur, respectively. Other nutritive modes such as facultative chemoautotrophy (e.g., *Strepyomyces* strain G26; Bell et al., 1988) and facultative methylotrophy (e.g., *Amycolatopsis methanolica*; Boer et al., 1990) have been observed among thermophilic actinobacteria. The diverse metabolic physiology facilitates the colonization of thermophilic actinobacteria in distinct topographical zones. Prevalence of thermophilic actinobacteria has been documented in sites ranging from the Desert Steppe Zone of Mongolia (Kurapova et al., 2012) to the subtropical area of Argentina (Carrillo et al., 2009) and hydrothermal vents to residential heating systems (Fink et al., 1971). Actinobacteria found in these environments are primarily fast growing and spore forming. The spores produced are of thermoduric type and are stable at higher temperatures for longer duration, even for days in some cases. This appears to provide an additional ecological advantage over other bacteria, making them easier to adapt back to their vegetative forms with the advent of favorable conditions.

### Systematics, taxonomy, and phylogeny

**Figure 1:**
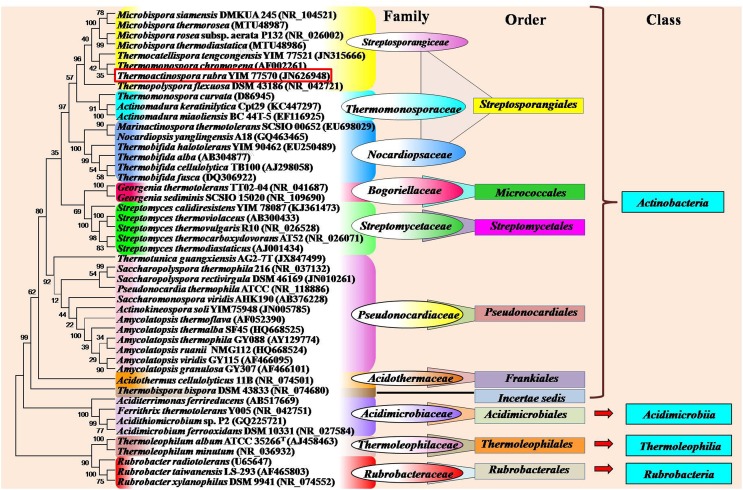
**Phylogram indicating the placement and relatedness of some thermophilic and thermotolerant actinobacterial strains belonging to four classes (***Actinobacteria***, ***Acidimicrobiia***, ***Rubrobacteria***, and ***Thermoleophilia***) of the phylum ***Actinobacteria*****. The numbers given at branch nodes indicate (%) bootstrap value. Phylogenetic tree was generated using Mega5.2 software with 1000 bootstrap replications. Bar 0.02 substitutions per 100 nucleotide positions.

Thermophilic and thermotolerant species exist in the diverse genera of phylum Actinobacteria (Table 1). Among them, the genera such as *Thermopolyspora, Thermomonospora, Thermotunica, Thermocatellispora, Thermobispora, Acidothermus, Acidimicrobium*, and *Thermoleophilum* include only thermophilic species, while other genera include both thermophilic and mesophilic species. All these genera belong to four classes such as *Actinobacteria, Acidimicrobiia, Rubrobacteria*, and *Thermoleophilia* of the phylum *Actinobacteria* (shown in Figure 1).

**Table 1:** **Thermophilic and thermotolerant actinobacterial species**.

**Actinobacteria**	**Growth conditions**		**Location of isolation**	**References**
	**Temperature (°C)**	**pH**		
*Microbispora siamensis* DMKUA 245^T^	25–50	–	Soil sample, Thailand	Boondaeng et al., 2009
*Georgenia sediminis* SCSIO 15020^T^	24–60	6–10	Sea sediment, Austria	You et al., 2013
*Actinokineospora soli* YIM 75948^T^	25–55	7–9	Soil sample, China	Tang et al., 2012
*Marinactinospora thermotolerans* SCSIO 00652^T^	10–55	6–9	Sea sediment, Northern South China	Tian et al., 2009
*Saccharomonospora viridis* SJ-21	35–60	7–10	Hot water spring, India	Jani et al., 2012
*Actinomadura miaoliensis* BC 44T-5^T^	22–55	7.0	Soil sample, Taiwan	Tseng et al., 2009
*Streptosporangium* sp.	–	–	Soil of Mongolia Desert Steppe Zone	Kurapova et al., 2012
*Streptomyces Calidiresistens* YIM 7808^T^	40–65	7.0	Hot spring sediment, South-west China	Duan et al., 2014
*Nocardiopsis yanglingensis* A18	25–55	6.5–8.5	Compost of button mushrooms	Yan et al., 2011
*Amycolatopsis ruanii* NMG112^T^	20–50	4–10	Soil sample	Zucchi et al., 2012
*A. thermalba* SF45^T^				
*A. granulosa* GY307^T^				
*Pseudonocardia thermophila* JCM3095	–	–	–	Yamaki et al., 1997
*Thermomonospora curvata* B9T	40–65	7.5–11	Composted stable manure	Chertkov et al., 2011
*Thermobifida fusca* (formerly named as *Thermomonospora fusca*)	35–53	10–11	–	McCarthy and Cross, 1984
*Thermotunica guangxiensis*	37–65	6–9	Mushroom residue compost, China	Wu et al., 2014b
*Thermopolyspora flexuosa* DSM 41386^T^	40–60	6–9	Soil from the Pamir Mountains	Krasilnikov and Agre, 1964
*Thermocatellispora tengchongensis*	28–58	6–8	Soil sample, South-west China	Zhou et al., 2012
*Saccharopolyspora thermophila* 216^T^	45–55	–	Soil sample, China	Lu et al., 2001
*Thermobispora bispora* R51^T^	50–65	–	Decaying manure, Berlin	Henssen, 1957
*Thermoleophilum album* ATCC 35263	45–70	6.5–7.5	Mud samples	Zarilla and Perry, 1984
*Acidothermus cellulolyticus* 11B	37–70	4–6	Acidic hot springs, Yellowstone National Park	Barabote et al., 2009
*Acidimicrobium ferrooxidans* TH3	45–50	2	Icelandic geothermal site	Clark and Norris, 1996
*Aciditerrimonas ferrireducens* IC-180^T^	35–58	2.0–4.5	Solfataric field, Japan	Itoh et al., 2011
*Acidithiomicrobium* sp.	50	3	Geothermal environments	Norris et al., 2011
*Ferrithrix thermotolerans* Y005T	43	1.3	Mine site, UK	Johnson et al., 2009
*Rubrobacter taiwanensis* LS-28	30–70 (optimum 60)	6–11	Lu-shan hot springs, Taiwan	Chen et al., 2004
*Rubrobacter radiotolerans*	46–48	7.0–7.4	Hot springs, Central Portugal	Ferreira et al., 1999
*R. xylanophilus*	60	7.5–8.0		

Monospore producing thermophilic actinobacteria belong to three major genera *Saccharomonopora, Thermomonospora*, and *Micromonopsora*. The genus *Saccharomonospora* was first described by Nonomura and Ohara (1971) for monosporic actinobacteria with cell wall type IV (meso-DAP, arabinose, and galactose), which includes mostly mesophilic actinobacteria except *Saccharomonospora xinjiangensis* (Jin et al., 1998) and *S. viridis*. The genus *Thermomonospora* was originally proposed only for thermophilic actinobacteria (Henssen, 1957), which comprised three thermophilic species *T. curvata, T. lineata*, and *T. fusca*. Only *T. curvata* could be maintained as pure culture among the three. Afterwards, one mesophilic actinobacterium (*T. mesophila*) was transferred from the genus *Actinobifida* to the genus *Thermomonospora* (Nonomura and Ohara, 1971). Consequently, some other *Thermomonospora* species such as *T. mesouviformis* (Nonomura and Ohara, 1974) and *T. curvata, T. alba, T. chromogena, T. fusca*, and *T. mesophila* (McCarthy and Cross, 1984) were identified. Later on, the *T. mesouviformis* was reassigned as a synonym of *T. alba* (McCarthy and Cross, 1984). One more species, *T. formosensis* (Hasegawa et al., 1986), was isolated and introduced into this genus. McCarthy (1989) described a total of six species (*T. curvata, T. alba, T. chromogena, T. fusca, T. mesophila*, and *T. formosensis*) in the ninth edition of Bergey's Manual of Determinative Bacteriology. Zhang et al. (1998) proposed a polyphasic taxonomy based classification system for the six *Thermomonospora* species. *T. formosensis* and *T. mesophila* were reclassified as *Actinomadura formosensis* and *Microbispora mesophila*, respectively. *T. alba* and *T. fusca* were transferred to the genus *Thermobifida* and named as *Thermobifida alba* and *Thermobifida fusca*, respectively (Zhang et al., 1998). The genus *Themomonospora* is now left with only two species (*T. curvata* and *T. chromogena*). However, *T. chromogena* (shown in red square in Figure 1) appears distantly from *T. curvata* on 16S rRNA tree. It shows close ribosomal gene sequence similarity with *Thermobispora bispora*. The detailed study of *T. chromogena* revealed the presence of total six rRNA operons (rrn) in the genome, among which, one operon (rrnB) shows sequence similarity with rRNA of *Thermobispora bispora*. The thermophilic actinobacterium *T. chromonogena* might have acquired this operon from *Thermobispora bispora* or other related microorganism through horizontal gene transfer (Yap et al., 1999). The species of *Thermobifida* genus produces single spore on dichotomously branched hyphae. This genus includes only four species (shown in Figure 1). Among them, *Thermobifida fusca* is well-studied, which produces a number of industrially important enzymes and other bioactive compounds.

Bisporic thermophilic actinobacteria are included into two genera (*Thermobispora* and *Microbispora*). A thermophilic actinobacterium, *Thermobispora bispora* [earlier known as *Microbispora bispora* (Lechevalier, 1965) and *Thermopolyspora bispora* (Henssen, 1957)] has been isolated from decaying manure in Berlin, Germany (Henssen, 1957), and described as a type species of the genus *Thermobispora* based on thermal preference, chemotaxonomic features, and ribotyping (Wang et al., 1996). The genus contains only single species *T. bispora* that belongs to the class *Actinobacteria* (Goodfellow et al., 2012). In recent years, a few thermotolerant species were identified belonging to the genus *Microbispora* (shown in Figure 1).

Oligospore forming thermophilic actinobacteria are majorly included in the genera *Thermopolyspora, Saccharopolyspora*, and *Streptomyces*. A thermophilic actinobacterium, *Thermopolyspora flexuosa*, is the only species of the genus *Thermopolyspora* (Krasilnikov and Agre, 1964), which forms a short chain of spores on sporophore. This species had been subjected to several reclassifications and subsequently assigned into different genera such as *Nocardia* (Becker et al., 1964; Lechevalier et al., 1966), *Micropolyspora* (Krasil'nikov et al., 1968), *Actinomadura* (Cross and Goodfellow, 1973; Lacey et al., 1978), *Microtetraspora* (Kroppenstedt et al., 1990), and later into the genus *Nonomuraea* (Zhang et al., 1998). Once again the taxonomic position of this actinobacterium has been reconsidered and transferred from the genus *Nonomuraea* to the genus *Thermopolyspora* and rechristened as *Thermopolyspora flexuosa* on the basis of 16S rRNA sequence, chemotaxonomy, morphological, and physiological properties (Goodfellow et al., 2005).

The genus *Saccharopolyspora* includes both mesophilic and thermophilic species. The thermophilic species such as *S. rectivirgula* [formerly named as *Micropolyspora faeni, Thermopolyspora polyspora* (Henssen, 1957), and *Thermopolyspora rectivirgula* (Krasilnikov and Agre, 1964)] has been isolated from moldy hay. It causes severe farmer's lung disease. Another species of thermophilic *Saccharopolyspora, S. thermophila* was isolated from a garden soil collected from the Xishan Mountain, Beijing (Lu et al., 2001). Goodfellow et al. (1987) isolated a number of thermophilic *Streptomyces* species from diverse habitats. *Streptomyces thermovulgaris* had been reported as the causative agent of bacteremia (Ekkelenkamp et al., 2004), which has been further designated as a synonym of *S. thermonitrificans* (Kim et al., 1999). Some other thermophilic *Streptomyces* such as *Streptomyces* sp. G26 (Bell et al., 1988), *S. thermoautotrophicus* (Gadkari et al., 1990), *S. thermocarboxydovorans*, and *S. thermocarboxydus* (Kim et al., 1998) have been reported to be carboxydotroph, which are capable of oxidizing the toxic carbon monoxide gas into innocuous CO_2_, thus, lowering its atmospheric concentration to safer levels.

Non-sporulating thermophilic actinobacteria belong to the genus *Rubrobacter* (Suzuki et al., 1988) which includes many thermophiles or radiotolerant thermophiles and mesophiles. A thermophilic and radiotolerant actinobacterium, *R. radiotolerans* was formerly described as *Arthrobacter radiotolerans* (Yoshinaka et al., 1973), which tolerates both gamma and UV radiations (Suzuki et al., 1988). The complete genome sequence of *R. radiotolerans* RSPS-4 has been recently annotated to elucidate the radiation resistant mechanism (Egas et al., 2014). Other thermophilic actinobacteria belonging to this genus are *R. xylanophilus* (Carreto et al., 1996), *R. taiwanensis* (Chen et al., 2004), *R. calidifluminis*, and *R. naiadicus* (Albuquerque et al., 2014). The non-sporulating genus, *Amycolatopsis* also includes a few thermophilic actinobacteria (shown in Figure 1). *Aciditerrimonas ferrireducens* (Itoh et al., 2011), *Acidithiomicrobium* sp. (Norris et al., 2011), *Ferrithrix thermotolerans* (Johnson et al., 2009) and *Acidimicrobium ferrooxidans* (Clark and Norris, 1996) are non-spore forming thermoacidophilic actinobacteria belonging to the class *Acidimicrobiia*. *Aciditerrimonas ferrireducens* exhibits both heterotrophic and autotrophic mode of nutrition. It is capable of reducing ferric ions to facilitate the autotrophic growth under anaerobic conditions, while the last two catalyze both the processes (dissimilatory oxidation of ferrous iron and reduction of ferric iron). *Acidimicrobium ferrooxidans* displays facultative autotrophic growth, which is capable of fixing atmospheric CO_2_ in the absence of organic matter, while *Ferrithrix thermotolerans* exhibits only heterotrophic mode of nutrition. Another thermoacidophilic actinobacterium, *Acidothermus cellulolyticus* 11B was isolated from hot-springs (Mohagheghi et al., 1986), which belongs to the order *Frankiales*. It produces a number of thermostable cellulases, among which, a cellulase (endoglucanases E1) shows higher thermostability and substrate specificity as compared to other actinobacterial cellulases (Thomas et al., 1995).

### Adaptation of thermophilic and thermotolerant actinobacteria

Thermotolerant/thermophilic actinobacteria have acquired diverse strategies for homeostasis such as comparatively higher GC content in their genomes, substitution of amino acids in proteins and contain specific components in the cell wall. Mostly thermophiles are known to incorporate comparatively higher quantity of charged amino acids (Asp, Glu, Arg, and Lys) than polar amino acids (Asn, Gln, Ser, and Thr) in their proteins (Suhre and Claverie, 2003). Same trend of increased content of charged amino acids except lysine was observed in the proteins of *Thermobifida fusca* (Lykidis et al., 2007). The genus *Corynebacterium* includes mostly mesophilic actinobacteria with the exception of *C. efficiens* which is capable to grow up to 45°C (Fudou et al., 2002). The comparatively high GC content may provide the thermotolerance to the *C. efficiens*. Amino acid substitution has also been noticed in the enzymes involved in the biosynthetic pathway of industrial valuable amino acids (glutamic acid and lysine) which enhances the production yield of amino acids, thereby adding an industrial importance to this actinobacterium (Nishio et al., 2003). Another thermotolerant actinobacterium, *Saccharomonospora xinjiangensis* contains specific phospholipid [unknown glucosamine-containing phospholipids (GluNU)] in the cell wall, which is considered to be involved in favoring the growth at high temperatures (45–50°C~; Jin et al., 1998). *Acidothermus cellulolyticus* belongs to the family *Acidothermaceae* and the order *Frankiales*, can grow optimally at 55°C and pH 5.5. It comes close to the genus *Frankia* on the phylogenetic tree constructed on the basis of the 16S rRNA (Normand et al., 1996), recA (Maréchal et al., 2000), and shc nucleotide sequences (Alloisio et al., 2005). The thermal adaptation in *A. cellulolyticus* may be attributed to the presence of higher GC content compared to the *Frankia* species. The inverse nucleotide preference for G and A at the first and third codon positions has also been observed. Moreover, the proteins contain repetitive patch of the amino acids (IVYWREL) as compared to proteins of *Frankia* species. The amino acid patch might provide thermostability to proteins of *Acidothermus cellulyticus* (Barabote et al., 2009).

### Characteristic features of thermophilic and thermotolerant actinobacteria

All thermophilic and thermotolerant actinobacteria except the genera (*Amycolatopsis, Rubrobacter, Ferrithrix, Acidothermus, Aciditerrimonas, Acidimicrobium*, and *Thermoleophilum*) are spore formers. Mostly they are non-acid fast, non motile, and aerobes except the genus *Amycolatopsis* which includes both aerobes and facultative anaerobes. All are Gram-positive with the exception of *Thermoleophilum* sp., *Ferrithrix* sp., and a species (*S. viridis*) of the genus *Saccharomonospora*. The accurate status of thermophilic actinobacteria has been validated only after the advent of polyphasic taxonomy. Cell wall (peptidoglycan) composition is one of the major feature of the genus specific classification. On the basis of amino acid and sugar contents, actinobacterial cell wall is grouped into four major types i.e., type-I [LL-DAP (diaminopimelic acid) and glycine], type-II [amino acids (meso-DAP and glycine) and sugars (arabinose and xylose)], type-III (meso-DAP with or without madurose), type-IV (meso-DAP, arabinose and galactose; Lechevalier et al., 1966), and other cell wall type V–X. The majority of the thermophilic actinobacteria have a cell wall type-III, while a few genera (*Saccharomonospora, Saccharopolyspora*, and *Amycolatopsis*) are known to contain cell wall type IV. Only one species of the genus *Streptomyces* has cell wall type-I. Other cellular components considered for chemotaxonomic classification include phospholipids, fatty acids, mycolic acid, menaquinones type, and GC content (% mol). The major respiratory menaquinones of thermophilic and thermotolerant actinobacteria are MK-9 variants. The presence of other menaquinones MK-8 (*Rubrobacter*) and MK-10 (*Thermobifida*) have also been reported (Goodfellow et al., 2012) in thermophilic actinobacteria.

### Ecological importance

Thermophilic and thermotolerant actinobacteria are known to possess unique metabolic rates and physical properties that prove to be beneficial in a variety of ecological roles.

#### Composting

Composting is a self-heating, aerobic, and biodegradation process that supplies humus and nutrients to the soil (Rawat and Johri, 2013). The composting involves the synergistic action of bacteria, actinobacteria, and fungi, wherein the actinobacteria proliferate in the later stages of composting. The predominance of thermotolerant actinobacteria is generally observed in thermobiotic condition generated by the preceding bacteria. During the initial stage of thermobiotic condition, the compost is colonized by thermotolerant actinobacteria (*Streptomyces albus* and *Streptomyces griseus*) and subsequently by the thermophilic actinobacteria (Goodfellow and Simpson, 1987). Actinobacteria genera such as *Streptomyces, Amycolatopsis, Microbispora, Cellulosimicrobium, Micrococcus, Saccharopolyspora, Micromonospora, Thermobispora, Thermomonospora, Thermobifida*, and *Planomonospora* were reported to be involved in the composting process. The composition of actinobacterial communities varies during various stages of composting (Xiao et al., 2011). They also suppress the growth of plant pathogens by secreting antibiotics along with the breakdown of organic matter which provides an additional advantage of using compost in order to enhance soil nutrients and also suppressing the development of plant diseases. Moreover, the addition of compost to contaminated soil enhances the bioremediation rates of pollutants such as polycyclic aromatic hydrocarbons, petroleum, pesticides, and heavy metals (Chen et al., 2015).

#### Antimicrobial activity

Thermotolerant actinobacteria such as *Streptomyces tauricus, S. toxytricini, S. coeruleorubidis, S. lanatus*, and *Streptosporangium* sp. have been found to inhabit the rhizosphere of many plants in the desert of Kuwait during the hot season (Diab and Al-Gounaim, 1985). The rhizosphere inhabiting actinobacteria exhibit antimicrobial activity, thus protect the plant from the attack of phytopathogens (Xue et al., 2013). Some thermotolerant actinobacteria isolated from the Himalayan Mountains, have also been shown to exhibit antagonistic activity against pathogenic bacteria and fungi. They include mostly *Streptomyces* species such as *S. phaeoviridis* and *S. griseoloalbus, S. viridogens*, and *S. viridogens*. The *S. phaeoviridis* and *S. griseoloalbus* exhibit antibacterial activity against both Gram-positive and Gram-negative bacteria, including methicillin resistant and vancomycin resistant strains of *Staphylococcus aureus*. The other two *Streptomyces* species (*S. viridogens* and *S. rimosus*) are capable of suppressing the growth of pathogenic fungi (*Fusarium solani, Rhizoctonia solani, Colletotricum falcatum*, and *Helminthosporium oryzae*), therefore, these *Streptomyces* species could be used as the bio-pesticides for agricultural production (Radhakrishnan et al., 2007).

#### Plant growth promotion

Actinobacteria secrete many volatile secondary metabolites which play significant roles in the suppression of plant diseases and the alleviation of biotic or abiotic stresses. Moreover, many actinobacteria species are known to secrete the iron chelating organic molecules such as siderophores which sequester the solubilized form of iron (Fe^+3^) and immobilize it in the rhizosphere of plants growing in the iron deficient soil. The siderophores modulate either the plant growth, directly or indirectly, by enriching the other plant beneficial microbes in the rhizosphere zone (Palaniyandi et al., 2013). Dimise et al. (2008) showed that a soil dwelling cellulolytic actinobacterium, *Thermobifida fusca* partakes in plant growth promotion by synthesizing the siderophore (fuscachelins) through non-ribosomal peptide biosynthetic pathways.

#### Nitrogen fixation

The *Frankia* and some non-*Frankia* actinobacteria have been shown to fix the atmospheric nitrogen (Gtari et al., 2012). A thermophilic actinobacterium, *Streptomyces thermoautotrophicus* which is an autotrophic carboxydotroph, has an unusual characteristic of nitrogen fixation (Ribbe et al., 1997). In this actinobacterium, the process of nitrogen fixation is coupled to the oxidation of carbon monoxide. The electrons generated during the oxidation process of CO reduce molecular oxygen into oxygen free radicals. The manganese-containing superoxide oxidoreductase oxidizes the formed free radicals into O_2_ and release electrons. The released electrons are further utilized by the enzyme nitrogenase in order to reduce N_2_ into ammonia. The notable feature of nitrogenase of *S. thermoautotrophicus* is its insensitivity to O_2_ and ${\text{O}}_{2}^{-}$ radicals. Furthermore, it also differs from other known nitrogenases in terms of protein structure and requirement of Mg^2+^ and ATP. Valdes et al. (2005) reported that some *Thermomonospora* species are also capable of fixing atmospheric nitrogen.

#### Hypersensitivity pneumonitis

Besides their beneficial activities, thermophilic actinobacteria such as *Saccharomonospora viridis* (Pati et al., 2009) and *Saccharopolyspora rectivirgula* (Pettersson et al., 2014) have been reported to cause severe respiratory diseases such as Farmer's lung and bagassosis. The Farmer's lung and bagassosis are a type of hypersensitivity pneumonitis (HP). The major cause of these allergic reactions is attributed to the exposure to moldy molasses, when densely colonized by spore-forming thermophilic actinobacteria.

## Alkaliphilic and alkalitolerant actinobacteria

The actinobacteria have long been known to thrive in soda lakes, salt alkaline lake, and alkaline soil. Their occurrence has also been observed in neutral environments. The alkalitolerant actinobacteria are capable of growing in the comparatively broader range of environments from neutral to alkaline pH. Alkaliphilic actinobacteria are, therefore, categorized into three major groups: alkaliphilic (grow optimally at pH 10–11), moderately alkaliphilic (grow in a pH range of 7–10) but show poor growth at pH 7.0, and alkalitolerant actinobacteria (grow in the pH range between 6 and 11; Jiang and Xu, 1993). Baldacci (1944) presented the first report on alkaliphilic actinobacteria. Thereafter, Taber (1960) isolated alkaliphilic actinobacteria from the soil. The occurrence of alkaliphilic and alkalitolerant actinobacteria has been reported from various habitats including deep sea sediment (Yu et al., 2013), alkaline desert soil (Li et al., 2006), and soda lakes (Groth et al., 1997). Mikami et al. (1982) studied the distinct chemotaxonomic patterns of cell wall of a total six alkaliphilic *Streptomyces* species [*Streptomyces caeruleus* ISP 5103 (reclassified as *Actinoalloteichus cyanogriseus*, Tamura et al., 2008), *S. alborubidus* ISP 5465 (reclassified as *Nocardiopsis alborubida*), and *S. autotrophicus* ISP 5011, *S. canescens* ISP 5001, *S. cavourensis* ISP 5300, and *S. hydrogenans* ISP 5586] which show optimum growth at pH 11.5. Among them, the first three contained meso-diaminopimelic acid. Subsequently, the taxonomic positions and applications of alkaliphilic actinobacteria in various fields have been described by Groth et al. (1997) and Duckworth et al. (1998).

### Physiology, characteristic, and taxonomic features of alkaliphilic and alkalitolerant actinobacteria

The alkaliphilic and alkalitolerant actinobacteria are known to occur in environments of high salinity (known as haloalkaliphiles or haloalkalitolerants) or in thermobiotic conditions (termed as alkalithermophile or alkalithermotolerants). Alkalithermophiles and alkalithermotolerant actinobacteria have also been isolated from saline habitats with their halophilic and halotolerance characteristic (Zenova et al., 2011). One such polyextremotolerant actinobacterium, *Microbacterium sediminis* has been isolated from deep sea that possesses the psychrotolerance, thermotolerance, halotolerance, and alkalitolerance attributes (Yu et al., 2013). Other reported polyextremophilic actinobacteria include alkaliphilic and thermotolerant actinobacteria [*Streptomyces alkalithermotolerans* (Sultanpuram et al., 2014) and *Georgenia satyanarayanai* (Srinivas et al., 2012)], thermophilic and alkalitolerant (*Streptomyces thermoalcalitolerans*; Kim et al., 1999), and haloalkaliphilic actinobacteria [*Nitriliruptor alkaliphilus* (Sorokin et al., 2009)]. They are either aerobes or microaerobes or facultative anaerobes. All alkaliphiles and alkalitolerants are Gram-positive. These exist as either halophiles or non-halophiles. Most alkaliphilic and alkalitolerant actinobacteria are non-motile and spore- or non-spore formers.

**Figure 2:**
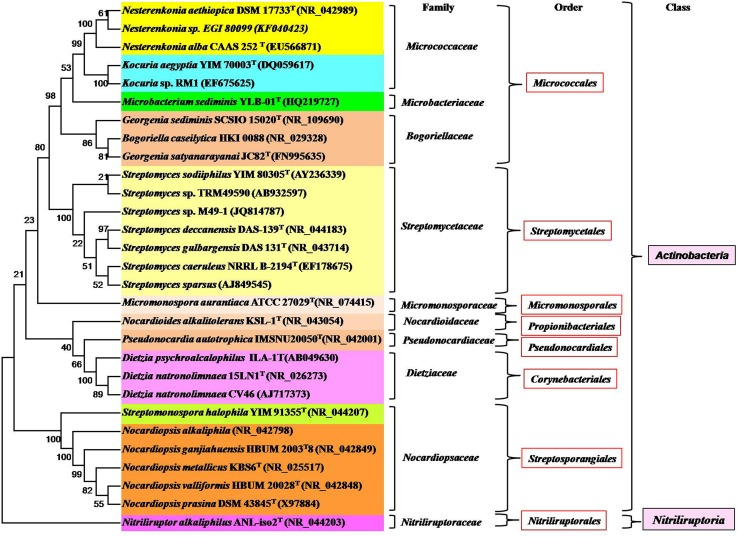
**Phylogram indicating the placement and relatedness of some alkaliphilic, alkalitolerant, alkalithermophilic and alkalithermotolerants actinobacterial strains belonging to two classes (***Actinobacteria*** and ***Nitriliruptoria***) of the phylum ***Actinobacteria*****. The numbers given at branch nodes indicate (%) bootstrap value. Bar 0.02 substitutions per 100 nucleotide positions.

Some alkaliphilic actinobacterial species belonging to the genus *Streptomyces* (Mikami et al., 1982), *Micromonospora* (Jiang and Xu, 1993), *Nocardioides* (Yoon et al., 2005), *Microcella* (Tiago et al., 2005), *Cellulomonas* (Jones et al., 2005), *Nesterenkonia* (Luo et al., 2009), *Streptosporangium* (Gurielidze et al., 2010), *Corynebacterium* (Wu et al., 2011b), *Georgenia* (Srinivas et al., 2012), *Nocardiopsis, Isoptericola, Nesterenkonia* (Ara et al., 2013), *Saccharomonospora* (Raut et al., 2013), *Saccharothrix* (Jani et al., 2014), and *Arthrobacter* (Kiran et al., 2015) have been isolated and well-characterized. Among them, the genus *Nocardiopsis* has been found to be prominent in alkaline environments (Ara et al., 2013). All the genera belong to the class *Actinobacteria* except the genus *Nitriliruptor* that belongs to the class *Nitriliruptoria* (shown in Figure 2). There are a few well-characterized alkalitolerant species such as *Citricoccus alkalitolerans* (Li et al., 2005), *Spinactinospora alkalitolerans* (Chang et al., 2011), and *Haloactinopolyspora alkaliphila* (Zhang et al., 2014) which proliferate in sites ranging from neutral to alkaline pH.

### Ecological significance

#### Microbial decomposition in hypersaline or haloalkaline sites

The microbial degradation of recalcitrant molecules takes place rapidly in the environment with acidic or neutral pH. However, the hypersaline and extreme haloalkaline conditions of lakes and mangroves limit most of the microbial hydrolytic activity on complex biomolecules such as cellulose, lignin, and chitin. Only haloalkaliphilic or haloalkalitolerant bacteria and actinobacteria are capable to proliferate and contribute in the decomposition of recalcitrant biopolymers in haloalkaline zones. A number of alkalitolerant or alkaliphilic actinobacteria have been isolated from mangrove, soda lakes and marine sediment. The two *Isoptericola* species i.e., *Isoptericola chiayiensis* (Tseng et al., 2011) and *Isoptericola rhizophila* (Kaur et al., 2014) were isolated from mangrove soil sample, Taiwan and rhizosphere of *Ficus benghalensis* (banyan tree) in Bhitarkanika mangrove forest, India, respectively. These two species are capable of hydrolyzing organic matter into simpler forms which are further assimilated by plants. The second most abundant biopolymer, chitin is produced by brine shrimp in bulk quantities in hypersaline soda lakes. Sorokin et al. (2012) showed the high prevalence of haloalkaliphilic chitinolytic bacteria and actinobacteria in hypersaline sediments and soda soils. The other chitinolytic actinobacteria species include *Isoptericola halotolerans, Nocardiopsis* sp., *Glycomyces harbinensis*, and *Streptomyces sodiiphilus* which are capable of degrading chitin completely and more rapidly than the bacterial population (Sorokin et al., 2012). Other alkaliphiles, *Nocardiopsis prasina* OPC-131 (Tsujibo et al., 2003), *Streptomyces* and *Nocardia* sp. (Bansode and Bajekal, 2006) are reported to display chitinolytic activity.

#### Chitin amendment

Chitin amendment is a soil management approach to suppress or inhibit the growth of plant pathogens or parasites. The addition of chitin enhances the pathogenic suppressiveness of soil (Kielak et al., 2013). This strategy not only involves the chitinolytic action of the soil or rhizosphere microflora but also induces desired changes in the metabolism of the endophytic microflora of plants (Hallmann et al., 1999). The *Arthrobacter* sp., *Corynebacterium aquaticum, Micrococcus luteus, Mycobacterium parafortuitum*, and other bacterial species were found during the chitin facilitated amendment of the soil and rhizophere zone of cotton plants (Hallmann et al., 1999). The microbial community has been found to change with the alteration of physical properties (pH and temperature) of soil. The enzyme chitinase produces short oligosaccharide chains and chitin derivatives which have various industrial applications. Besides biotechnological applications, the chitinases that are particularly active at high pH find application in plant pathogen suppression by hydrolyzing the cell wall component (chitin) of fungi, thereby inhibiting the fungal growth and spread of infection. The alkalistable chitinase producing *Isoptericola jiangsuensis* (Wu et al., 2011a) and *Nocardioides* sp. (Okajima et al., 1995) can be applicable for such soil amendment practices. The amendment of chitin with apatite has also been found to sequester the metals in marine sediments (Kan et al., 2013).

#### Biotransformation

The nitriles (RC≡N) are organic compounds, synthesized by chemical methods (ammoxidation, hydrocyanation, and dehydration of amides and oximes) or biologically produced by anaerobic degradation of amino acids (Harper and Gibbs, 1979). The cyanogenic plants also release nitrile compounds in the environment (Vetter, 2000). The nitriles are commonly used in the synthesis of other useful organic compounds or manufacturing of rubber (gloves) and super glue. Moreover, the selective hydrolysis or reduction of nitriles yields valuable compounds such as amides, acids, and amines. Despite their various uses, nitriles cannot be easily degraded and are known to persist for longer periods in the environment, causing toxic or hazardous effects on biological systems, therefore, nitriles have to be metabolized into non-toxic forms. The two enzymatic pathways [nitrile hydrolase/amidase (two steps) and nitrilases (single step)] are reported to be involved in the conversion of nitriles into carboxylic acid and ammonia. Some nitrile degrading bacteria, actinobacteria, and fungi have been isolated and characterized. Most of the well-known nitrile degraders are neutrophiles. Sorokin et al. (2007) showed that a microbial consortium could degrade nitriles completely. This consortium consists of an actinobacterium (*Nitriliruptor alkaliphilus* ANL-iso2^T^) and a bacterium (*Marinospirillum* sp. strain ANL-isoa). *Nitriliruptor alkaliphilus* ANL-iso2^T^ is an obligate alkaliphile and moderately salt-tolerant which plays a major role in the hydrolysis of isobutyronitrile (iBN; Sorokin et al., 2009). This actionobacterium has a nitrile hydratase/amidase pathway to metabolize isobutyronitrile (iBN) into isobutyroamide, isobutyrate and ammonia which are further scavenged by *Marinospirillum* sp. strain ANL-isoa. *Nitriliruptor alkaliphilus* ANL-iso2^T^ is also capable of utilizing propionitrile (C3), butyronitrile (C4), valeronitrile (C5), and capronitrile (C6) as carbon and nitrogen source, thus, indirectly cleaning the environment. This strain can, therefore, be applied as a potential candidate for bioremediation or other environmental biotechnological purposes.

#### Bioweathering

Weathering is a disintegration process of rock constituents into smaller fragments. These components are further broken down into mobilized forms of essential nutrients (e.g., P and S) and elements (e.g., Na, K, Mg, Ca, Mn, Fe, Cu, Zn, Co, and Ni). The essential nutrients and elements are brought into crop lands or fields through wind or water. Microbial populations (bacteria and actinobacteria) occupying the rock zones show high resistance to radiations, desiccation and limited nutrient conditions. The filamentous microbes are capable of enhancing the weathering process as they penetrate through the rocks by the growing mycelia. The *Streptomyces* species are most commonly observed in rock weathering sites, since they have filamentous structure and are capable of growing as oligotroph (Cockell et al., 2013). They have a great efficiency to utilize the recalcitrant organic matter and form anthrospore under water stress. Cockell et al. (2013) reported that the indigenous microbial population of Icelandic volcanic rocks includes *Arthrobacter, Knoellia, Brevibacterium, Rhodococcus*, and *Kribbella* species. The investigation of the altered stones and monuments in the Mediterranean basin also revealed the presence of actinobacterial species which involved in the weathering of stones and monuments. These species belong to the three genera *Geodermatophilus, Blastococcus*, and *Modestobacter* of the family *Geodermatophilaceae* (Urzì et al., 2001). Similarly, other actinobacterial species such as *Nocardioides, Kibdelosporangium* (Abdulla, 2009), *Arthrobacter*, and *Leifsonia* (Frey et al., 2010) are known to accelerate the weathering process. Furthermore, some other actinobacteria capable of carrying out withering of rocks are also alkalitolerant such as [*Isoptericola nanjingensis* H17T (Huang et al., 2012) and *Arthrobacter nanjingensis* A33T (Huang et al., 2015)] and have been isolated from soil samples of Nanjing, China.

#### Plant growth promotion

Actinobacteria are well-known to exhibit antimicrobial and insecticidal properties and help in suppression of plant pathogenesis, thereby indirectly promoting plant growth. They also make iron available to plants for their growth (Francis et al., 2010). The plants and microbes can take up iron only in its reduced form (Fe^+2^), while the iron exists as oxidized form (Fe^+3^) in alkaline soils. Alkaliphilic actinobacteria reduce the iron (from Fe^+3^ to Fe^+2^ forms) and make it into soluble form which can be assimilated by plants and microbes for their growth (Valencia-Cantero et al., 2007). These actinobacteria are also capable of solubilizing phosphorus in alkaline conditions as solubility of phosphorus decreases in acidic or alkaline soils (Palaniyandi et al., 2013). An alkaliphilic strain, *Kocuria rosea* HN01 reduces Fe^+3^ into the soluble form (Fe^+2^), thus, making the iron available to plants growing in the alkaline soil (Wu et al., 2014a).

#### Humic acid reduction

The oxidation and reduction of humic acid have a significant importance during the anaerobic biotransformation of organic and inorganic pollutants. The quinone moieties of humic acid act as center for oxido-reductive reactions (Lovley et al., 1996). The oxidized form of humic acid accepts electrons released from mineralization of organic pollutants. In addition, the reduced form of humic acid is also involved in biotransformation by reducing insoluble pollutants (oxidized) to soluble form (reduced). An alkaliphilic actinobacterium, *Corynebacterium humireducens* is capable of carrying out such biotransformation and catalyzes the reduction of the humic acids (Wu et al., 2011b) as well as the reduction of a quinone into hydroquinone. The hydroquinone speeds up the process of mineralization of pollutants such as 2,4-dichlorophenoxy acetic acid (Wang et al., 2009). The reduced humic acid could further be used to reduce the insoluble Fe^+3^ into soluble Fe^+2^ ions making them available for plant assimilation.

## Applications of thermophilic and alkaliphilic actinobacteria

**Figure 3:**
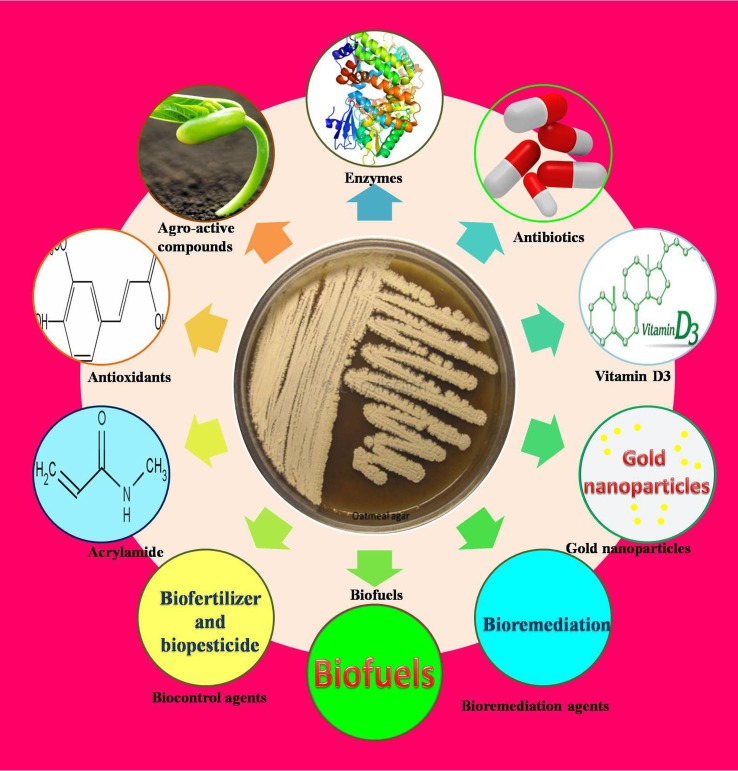
**Potential applications of Industrial thermophilic and alkaliphilic actinobacteria**.

Thermophilic and alkaliphilic actinobacteria are useful in bioremediation, gold nanoparticle synthesis, biofertilizers and biopesticides (Figure 3). In addition, they produce novel bioactive compounds and enzymes with commercial applications.

### Synthesis of gold nanoparticles

The prokaryotes (bacteria and actinobacteria) as well as eukaryotes (algae, fungi, and yeast) have been currently being explored for the manufacturing of nanoparticles. The mechanism of gold particle synthesis involves the reduction of Au^3+^ by microbes when they are incubated with gold chloride (Beveridge and Murray, 1980). They synthesize nanoparticles either intracellularly or extracellularly. Among them, the use of prokaryotes is preferred because of their capability to tolerate high concentration of metal (Silver, 2003), leading to the production of a higher yield of nanoparticles. Moreover, the synthesis of nanoparticles by actinobacteria has an additional advantage of polydispersity property which prevents self-aggregation of nanoparticles (Ahmad et al., 2003a). The synthesis of gold nanoparticles by *Thermomonospora* sp. (Ahmad et al., 2003a) and alkalitolerant actinomycete *Rhodococcus* sp. (Ahmad et al., 2003b) was studied. The gold particles find various applications in diagnostics, therapeutic, and catalytic purposes.

### Bioremediation of hydrocarbon contaminated sites

The thermophilic actinobacteria decompose a large number of biomolecules (lignin, cellulose, and hemicellulose) and recycle the nutrient back into soil which enhances the soil productivity. The process of biodegradation of interactive complex substrates necessitates actinobacteria to secrete a range of extracellular hydrolytic and oxidative enzymes. The rapid hyphal colonization and enzyme secretion enable them as being a good candidate for bioremediation process. Moreover, they are capable of metabolizing recalcitrant polymers (hydrocarbons, xenobiotic, and toxic pesticides), plastics, and rubber. Tseng et al. (2007) isolated several plastic degrading actinobacterial species belonging to the genera (*Actinomadura, Microbispora, Streptomyces*, and *Saccharomonospora*). These actinobacteria degrade various biodegradable polyesters such as poly(ethylene succinate) (PES), poly(e-caprolactone) (PCL), poly(D-3-hydroxybutyrate) (PHB), poly(tetramethylene succinate) PTMH, poly(L-lactide) (PLA), and terephthalic acid, and reduce their environmental impacts. A few other thermophilic actinobacteria are reported to act on polymer (rubber) and produce valuable chemicals such as carbonyl carbon atoms (aldehydes and ketone) and bifunctional isoprenoid species (Table 2). The toxic organic compounds include phenol and nitriles such as acrylonitrile and adiponitrile which are hazardous to human health. These harmful chemicals need to be degraded. Some thermophilic actinobacteria (listed in Table 2) are capable of metabolizing these lethal chemicals into non-toxic form by producing various enzymes such as phenol hydroxylase, polyphenol oxidase, catechol 2,3 dioxygenase, laccase, peroxidase, and nitrile converting enzymes (amidases, nitrilases, and nitrile hydratases). The pentachlorophenol is an organochlorine compound which works as a broad spectrum biocide and is used mainly in sawmills to preserve the woods. The soil and water resources of an area surrounding sawmills are contaminated with the chlorophenols causing hazardous effects on biological systems. The chlorophenols, therefore, need complete degradation. The *Saccharomonospora viridis* isolated from mushroom compost is capable of hydrolyzing this phenolic compound into non-toxic form (Webb et al., 2001).

**Table 2:** **List of thermophilic and alkaliphilic actinobacteria degrading plastics, rubber and organic pollutants**.

**Actinobacteria strains**	**Substrate degraded**	**References**
**PLASTICS DEGRADATION**		
*Actinomadura miaoliensis* BC 44T-5^T^	PHB	Tseng et al., 2009
*Actinomadura keratinilytica* T16-1	PLA	Sukkhum et al., 2012
*Thermobifida fusca*	Terephthalic acid	Kleeberg et al., 1998
*Thermobifida alba* AHK119	Terephthalic acid.	Hu et al., 2010
*Microbispora rosea* subsp. *aerata* IFO 14046	PTMH and PCL	Jarerat and Tokiwa, 2001
*Microbispora rosea* subsp. *aerata* IFO 14047		
*Excellospora japonica* IFO 144868		
*E. viridilutea* JCM 339		
*Streptomyces* sp. strain MG	PTMH and PCL	Tokiwa and Calabia, 2004
*Streptomyces thermoviolaceus* subsp. *thermoviolaceus* 76T-2	PCL	Chua et al., 2013
*Streptomyces bangladeshensis* 77T-4	PHB	Hsu et al., 2012
*Dietzia* sp. Strain GS-1	Disodium terephthalate	Sugimori et al., 2000
**RUBBER DEGRADATION**		
*Streptomyces* strain La 7	Latex and natural rubber	Gallert, 2000
*Actinomadura nitritigenes*	Poly(cis-1,4-isoprene)	Ibrahim et al., 2006
*Nocardia farcinica*		
*Thermomonospora curvata*		
**ORGANIC POLLUTANTS DEGRADATION**		
*Streptomyces setonii* strain ATCC 39116	Phenol and benzoate	An et al., 2000
*Pseudonocardia thermophila* JCM3095	Acrylonitrile	Yamaki et al., 1997
*Kocuria rosea* HN01	DDT (1,1,1-trichloro-2,2-bis(4-chlorophenyl) ethane)	Wu et al., 2014a
*Dietzia natronolimnaea* JQ-AN	Aniline	Jin et al., 2012
*Georgenia daeguensis*	4-Chlorophenol	Woo et al., 2012
*Nocardioides* sp.	2,4-Dichlorophenol and 2,4,5-trichlorophenol	Maltseva and Oriel, 1997
*Dietzia* sp. Strain DQ12-45-1b	Petroleum hydrocarbons and crude oils	Wang et al., 2011
*Dietzia cinnamea* P4	Petroleum hydrocarbons	Weid et al., 2007
*Dietzia* sp. PD1	Azo dyes	Das et al., 2015
*Dietzia* sp. E1	Long chain *n*-alkane	Bihari et al., 2010
*Dietzia* sp. *H0B*	Prestige oil spill	Alonso-Gutierrez et al., 2011

A number of alkalitolerant and alkaliphilic actinobacteria have been reported to mineralize the hydrocarbon and other pollutants. The *Dietzia* species were found to have organic pollutant degradability and produce biosurfactants or bioemulsifiers by degrading n-alkanes (Nakano et al., 2011). The biosurfactants can be used in pharmaceuticals, detergents, textiles, and cosmetics. The species of other genera have also been reported to degrade hydrocarbons (listed in Table 2). A biofilm isolated from hypersaline liquids, has been shown to remove the hydrocarbon pollutants (60–70% of crude oil, pure n-hexadecane, and pure phenanthrene; Al-Mailem et al., 2015). The two alkalitolerant actinobacteria such as *Kocuria flava* and *Dietzia kunjamensis* along with other bacterial community was reported in the biofilm. A biofilm is densely packed microbial community, formed by irreversible organization, cooperation, and secretion of polymers which facilitate the adherence of microbes to the substrates and hasten the process of biodegradation of toxic compounds. The alkaliphilic and alkalitolerant actinobacteria are known to play a role in bioremediation of hydrocarbon and other organic contaminants are listed in Table 2.

### Bioleaching

Bioleaching is a process of extracting the metals from ores. The occurrence of alkaliphiles is comparatively less than acidophiles in metal leaching sites. The two alkaliphilic actinobacteria such as *Nocardiopis* sp. (Kroppenstedt, 1992) and *Nocardiopsis metallicus* strain *KBS6*^T^ (Schippres et al., 2002) have a tendency to leach metals from the alkaline slag dump, could be applied in the process of metal extraction in alkaline sites.

### Bioremediation of radionuclides contaminated sites

The nuclear power plants generate huge amount of radioactive wastes (radionuclides) which contaminate the land areas and water resources e.g., lakes and rivers. The radionuclides contaminated sites contain other toxic compounds as well such as heavy metals (e.g., mercury) and toxic hydrocarbons. Exposures to these lethal compounds cause cancer, birth defects, and other abnormalities. Conventionally, the chemical (solvent extraction and chemical oxidation) or physical remediation (soil washing and soil vapor extraction) methods are employed to extract these hazardous pollutants. However, these methods are quite less efficient and expensive. The microbial remediation has been found to be cost effective with high efficacy and prevents spreading of radioactive wastes over a wider area. However, the radionuclides are highly unstable and disintegrate spontaneously to emit energy in the form of harmful radiations, which act as a principle factor to limit the use of bioremediation. Since most of the microbial population is sensitive to radiations and other stresses which necessitates to search and use of radiation resistant microbes for removal or oxidation of toxic metals (Gholami et al., 2015). Some alkaliphilic (*Kocuria rosea* MG2) and alkali tolerant actinobacterial species [*Kineococcus radiotolerans* (Phillips et al., 2002), *Rubrobacter taiwanensis* (Chen et al., 2004), *Microbacterium radiodurans* (Zhang et al., 2010), and *Cellulosimicrobium cellulans* UVP1 (Gabani et al., 2012)] are resistant to lethal radiations and can sustain under harsh conditions, thus, could be potential candidates for this purpose.

### Biocontrol agent

Actinobacteria are known to improve the quality of compost and increase its nutrient content. In addition, they also reduce the odor of compost as they are able to completely digest the organic matter present in compost (Ohta and Ikeda, 1978). The thermophilic actinobacteria (*Streptomyces* sp. No. 101 and *Micromonospora* sp. No. 604) have been shown to degrade yeast debris completely and deodorize the compost (Tanaka et al., 1995). Mansour and Mohamedin (2001), reported that the *Streptomyces thermodiastaticus* produced many extracellular enzymes involved in the cell lysis of pathogenic fungi like *Candida albicans*. Some thermophilic actinobacteria are capable of suppressing plant diseases, thereby promoting good health of crop plants which leads to increase in crop yield (Iijima and Ryusuke, 1996), therefore, these thermotolerant actinobacteria could be used as alternative to commercial pesticides.

### Bioactive compounds production

Actinobacteria are a rich source of clinically important compounds, most importantly the compounds having antitumor, antimicrobial and immunosuppressive activities (Pritchard, 2005). They are the largest antibiotic producers among all microbes, and produce approximately 55% of the total known antibiotics (Raja and Prabakarana, 2011). Among these, 75% were discovered from *Streptomyces* and remaining 25% were from non-*Streptomyces* species. The bioactive compounds discovered till date are largely of mesophilic origins. A very few natural compounds have been reported from thermophilic and alkaliphilic actinobacteria (shown in Table 3). Most of the antibiotics of mesophilic origin are thermolabile that is they require low temperature to sustain their effectiveness, which may be problematic for longer storage and shipping practices. Routine use of such antibiotics leads to their degradation due to repeated freezing and thawing (Eisenhart and Disso, 2012). Some antibiotics are water insoluble (Stone, 1960) and organic solvent labile, therefore, need to be dissolved in warm water to improve their solubilization; this necessitates exploring thermophilic actinobacteria that produce thermostable alternatives to currently available antibiotics.

**Table 3:** **List of bioactive compounds produced by thermophilic and alkaliphilic actinobacteria**.

**Actinomyces isolates**	**Bioactive compounds**	**Activity**	**References**
**THERMOPHILIC ACTINOBACTERIA**			
*Excellospora viridilutea* SF2315 [reclassified as *Actinomadura viridilutea* (Zhang et al., 2001)]	SF2315A and B	Antibacterial	Sasaki et al., 1988
*Streptomyces thermophilus*	Thermomycin	Antibacterial	Schone, 1951
*Thermomonospora* sp.	T-SA-125	Antibacterial	Dewendar et al., 1979
*Streptomyces refuineus* subsp. *thermotolerans*	Anthramycin	Antitumor Antimicrobial	Hu et al., 2007
*Microbispora aerata*	Diketopiperazine	Neuroprotective agents	Ivanova et al., 2013
*Microbispora aerata*	Microbiaeratin	Antiproliferative and cytotoxic drug	Ivanova et al., 2007
*Marinactinospora thermotolerans*	β-Carboline and indolactam alkaloids	Antimalarial	Huang et al., 2011
**ALKALIPHILIC ACTINOBACTERIA**			
*Streptomyces werraensis*	Erythromycin	Antibacterial	Sanghvi et al., 2014
*Nocardiopsis dassonvillei* WA52	WA52-A Macrolide	Antifungal	Ali et al., 2009
*Streptomyces* sp. No. 1543	Antimycin A	Antifungal	Sato et al., 1985
*Streptomyces* sp. DPTTB16	4′Phenul-1-napthyl-phenyl acetamide	Antifungal	Dhanasekaran and Panneerselvam, 2008
*Streptomyces griseus* Var. *autotrophicus*	Faeriefungin	Antimicrobial and insecticidal activity	Nair et al., 1989
*Streptomyces* strain	Pyrocoll	Antiparasitic Antitumor	Dietera et al., 2003
*Nocardiopsis* sp.	Griseusin D	Anticancer	Li et al., 2007a
*Nocardiopsis alkaliphila* YIM-80379	Nocardiopyrones A and B	Antimicrobial	Wang et al., 2013
*Nocardiopsis terrae* YIM 90022	Quinolone alkaloid and N-acetyl-anthranilic acid	Antimicrobial	Tian et al., 2014

### Synthesis of pharmaceutical valuable compounds

Actinobacteria synthesize a large array of secondary metabolites (antioxidant, anti-inflammatory compounds, and clinically important enzymes; shown in Table 4). The antioxidants produced by the thermophilic and alkaliphilic actinobacteria are melanin, ferulic acid, and canthaxanthin. These antioxidants have multiple uses in the medical field, which have been used in the treatment of cancer, heart diseases and neurodegenerative disorders such as Alzheimer and Parkinson's diseases. Ferulic acid is a component of lignin, which is linked via the ester bonds to the polysaccharides (Scalbert et al., 1985). Ferulic acid is formed upon hydrolysis of lignin by feruloyl esterase (Huang et al., 2013). Apart from functioning as antioxidants, ferulic acid can also be used as a precursor for the synthesis of vanillin (food aromatic compounds), polymers, epoxides, and aromatic compounds (alkylbenzenes, protocatechuic acid-related catechols, guaiacol, and catechol; Rosazza et al., 1995). An alkalitolerant, *Dietzia* sp. K44 produces canthaxanthin (diketocarotenoid) which has comparatively more antioxidant property than β-carotene and zeaxanthin. Canthaxanthin is naturally produced in animal and plant tissues to scavenge the free radicals (Venugopalan et al., 2013). Another important secondary metabolite, carotenoids (tetraterpenoid) is produced by *Dietzia natronolimnaea* HS-1 (Gharibzahedi et al., 2014). Carotenoids can be used as vitamin A precursor, free radicals scavenger and enhancer of the *in vitro* for the production of antibodies. *Dietzia natronolimnaea* HS-1 also produces canthaxanthin which was tested in the formulation of stable nanoemulsion (NE). The nanoemulsion system is a method to solubilize the hydrophobic antitumor compounds, which uses 2-hydroxypropyl-b-cyclodextrin (HP-β-CD) to formulate the water based drugs. The stability of NE was enhanced by mixing canthaxanthin with HP-β-CD to yield the stable inclusion complex. The stable NE has imperative therapeutical applications (Gharibzahedi et al., 2015).

**Table 4:** **Pharmaceutically valuable compounds and enzymes produced by thermophilic and alkaliphilic actinobacteria**.

**Biological compounds**	**Actinobacteria isolates**	**Uses**	**References**
**ANTIOXIDANTS AND ANTI-INFLAMMATORY COMPOUNDS**			
Melanin	*Streptomyces lusitanus* DMZ-3	Cytotoxic compound	Madhusudhan et al., 2014
	*Streptomyces* sp.	Antioxidant	Quadri and Agsar, 2012
	*Streptomyces species* D5	Neurogenic disorder treatment	Diraviyam et al., 2011
Ferulic acid	*Thermobifida fusca* PU13-13	Antioxidant	Huang et al., 2013
		Anti-inflammatory	
Canthaxanthin	*Dietzia* sp. K44	Antioxidant	Venugopalan et al., 2013
		Feed additive	
		Cosmetics	
Carotenoids	*Dietzia natronolimnaea* HS-1	Antioxidant	Gharibzahedi et al., 2014
		Feed additive	
		Cosmetics	
**PHARMACEUTICALLY VALUABLE ENZYMES**			
Vitamin D3 hydroxylase	*Pseudonocardia autotrophica*	Bone metabolism	Fujii et al., 2009
		Immunity	
		Cell growth regulators	
Fibrinolytic enzyme	*Streptomyces* sp. MCMB-379	Blood clot dissolution	Chitte et al., 2011
	*Streptomyces megasporus* SD5		Chitte and Dey, 2002
Recombinant Asparaginase	*Streptomyces thermoluteus* subsp. *fuscus* NBRC 14270	Leukaemia treatment	Hatanaka et al., 2011a
L-Glutaminase	Alkaliphilic *Streptomyces* sp. SBU1	Leukaemia treatment	Krishnakumar et al., 2011
Ribonuclease	Alkaliphilic *Streptomyces* sp. M49-1	Antiviral	Demir et al., 2013
PrP^Sc^-degrading enzyme keratinase	*Nocardiopsis* strain TOA-1	Antiprion drug	Mitsuiki et al., 2010
Recombinant X-prolyl-dipeptidyl aminopeptidases (XDAP)	*Streptomyces thermoluteus* subsp. *fuscus* NBRC 14270	Antidiabetic agents	Hatanaka et al., 2011b
	*S. thermocyaneoviolaceus* NBRC 14271		
**ALDOSE REDUCTASE INHIBITOR**			
YUA001	*Corynebacterium* sp. YUA25	Antidiabetic agents	Bahn et al., 1998
**DNA POLYMERASE INHIBITORS**			
Topostatin	*Thermomonospora alba* Strain No. 1520 III [reclassified as *Thermobifida alba* (Zhang et al., 1998)]	Antiviral	Suzuki et al., 1998
Isoaurostatin	*Thermomonospora alba* [reclassified as *Thermobifida alba* (Zhang et al., 1998)]	Antiviral	Suzuki et al., 2001

Some clinically important enzymes have also been reported from thermophilic actinobacteria such as *Streptomyces* sp. (Chitte and Dey, 2002; Chitte et al., 2011) which have been shown to produce fibrinolytic enzymes. Fibrinolytic enzymes dissolve the blood clot (fibrin) into smaller peptides and decrease the blood viscosity, and can be used for reducing the risk of arteriosclerosis, heart attack, and stroke. Asparaginase is a well-known anticancer enzyme which inhibits the growth of uncontrolled rapidly dividing cells by hydrolyzing the amino acid asparagine which is required by the rapidly proliferating cancer cells. Hatanaka et al. (2011a) cloned and expressed the asparaginase of *Streptomyces thermoluteus* subsp. *fuscus* NBRC 14270 Another pharmaceutically valuable enzyme, X-prolyl-dipeptidyl aminopeptidase (XDAP) is known to be produced by thermophilic *Streptomyces* sp. (Hatanaka et al., 2011b), which acts on proline rich proteins and produces short peptides. These peptides act as inhibitors of dipeptidyl peptidase-4 (DPP-IV) and can regulate the blood sugar levels as DPP-IV degrades glucagon like protein-1 (GLP-1) which regulates insulin production and lowers the blood sugar level. Thus, it could be used along with GLP-1 to treat diabetes (Hatanaka et al., 2011b). Another clinically important enzyme, vitamin D3 hydroxylase converts cholecalciferol (VD_3_)to its biologically active form calcitriol [1α,25(OH)_2_VD_3_]. The cholecalciferol (VD_3_) is an inactive form, synthesized from 7-dehydrocholesterol in the epidermal layer of skin through electrocyclic reaction on irradiance of ultraviolet. The bioconversion of VD_3_ is a two step process, first it gets converted to calcidiol [25(OH)VD_3_] by P450 in the liver, and then subsequently hydrogenated to calcitriol by P450 in the kidney. The calcitriol is a physiologically active form of vitamin D, which is involved in the regulation of calcium and phosphate concentration in the blood plasma. This calcidiol and calcitriol can be artificially synthesized from cholesterol by a multistep chemical process, but the yield is very low. There is, thus, a need of an enzyme that can catalyze the hydrogenation of VD_3_ in a single step. Fujii et al. (2009) showed that *Pseudonocardia autotrophica* produces vitamin D3 hydroxylase catalyzing the conversion of VD3 into calcitriol, thus, could be used in the production of vitamin D (Fujii et al., 2009). Another important enzyme, aldose reductase catalyzes the conversion of glucose into sorbitol through polyol pathway. The high accumulation of sorbitol causes diabetes and other complications like retinopathy and neuropathy. An inhibitor YUA001 was identified from alkaliphilic *Corynebacterium* sp., that acts as a potent inhibitor of aldose reductase (Bahn et al., 1998). The two thermophilic species, *Thermomonospora alba* (Suzuki et al., 2001) and *Thermobifida alba* (Suzuki et al., 1998) produce compounds such as topostatin and isoaurostatin, respectively. These two compounds act as inhibitors of DNA topoisomerase and interfere with cellular processes like replication, transcription and translation of viruses, and therefore, could function as potential antiviral compounds.

### Industrially important enzymes

**Figure 4:**
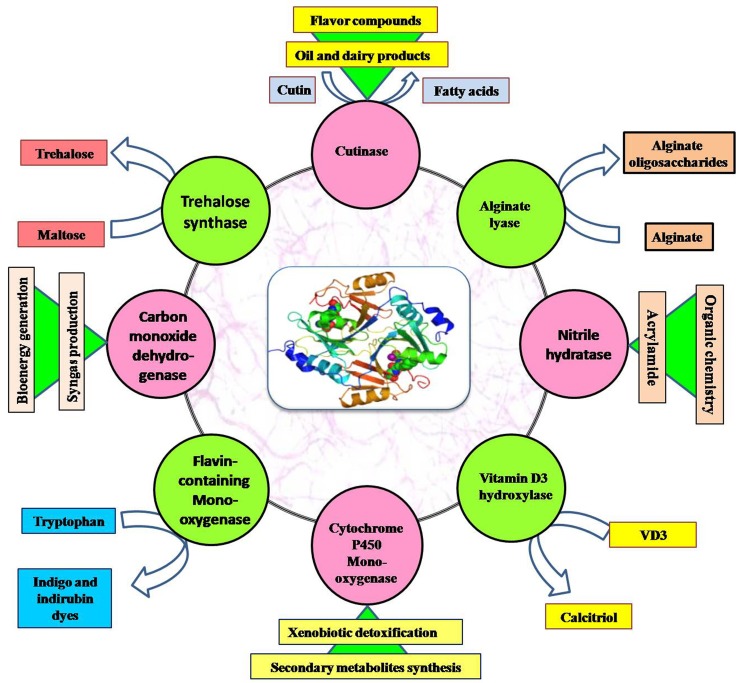
**Application of important enzymes produced by thermophilic and alkaliphilic actinobacteria**.

Other than the listed uses, thermophilic and alkaliphilic actinobacteria produce a number of enzymes (amylase, proteases, lipase, cellulase, xylanase, inulinase, dextranase, and keratinase; Table 5) which are being produced commercially and used in industries all over the world (shown in Figure 4). Some important actinobacterial enzymes are briefly described below.

**Table 5:** **Commercially relevant enzymes produced by thermophilic and alkaliphilic actinobacteria and their potential uses**.

**Enzyme**	**Actinobacteria strains**	**Optimum temperature and pH**	**Industrial applications**	**References**
Amylase	*Thermomonospora viridis* TF-35	60°C and 6.0	Detergent	Takahashi et al., 1992
			Baking	
			Paper and pulp	
	*Thermomonospora curvata*	65°C and 5.5–6.0	Textile industry	Glymph and Stutzenberger, 1977
Protease	*Saccharomonospora viridis SJ-21*	70°C and pH 9	Detergents	Jani et al., 2012
			Pharmaceutical	
	*Nocardiopsis prasina* HA-4	55°C and pH 7–10	Leather	Ningthoujam et al., 2009
			Brewing	
Keratinase	*Actinomadura keratinilytica* strain Cpt29	70°C and pH 10	Leather industry	Habbeche et al., 2014
	*Thermomonospora curvata*	65°C and pH 6	Pharamaceutical uses	Stutzenberger, 1971
Xylanase	*Thermomonospora fusca*	60°C and 7.0	Paper and pulp	McCarthy et al., 1985
			Baking	
	*Kocuria* sp. RM1	30–85°C and pH 4.5–9	Animal feed	Krishna et al., 2008
	*Streptomyces* sp. Ab 106	60°C and pH 9.0		Techapun et al., 2002
Acetylxylan esterase	*Thermobifida fusca* NTU22	80°C and 8.0	Paper and pulp	Yang and Liu, 2008
Dextranase	*Streptomyces* sp. NK458	60°C and 9.0	Sugar mills	Purushe et al., 2012
Nitrile hydratase	*Pseudonocardia thermophila* JCM 3095	Thermostable (50–80°C)	Acrylamide production	Martinez et al., 2014
Laccase	*Thermobifida fusca* BCRC 19214	Stable at 50°C and pH 10.0	Waste treatment	Chen et al., 2013
			Textile dye treatment	
Carbon monoxide dehydrogenase	*Streptomyces* sp. strain G26	69°C	Bioenergy generation	Bell et al., 1988
			Biofilters	

#### Amylase

A starch hydrolyzing process yields oligosacchharides and other simpler sugars (glucose, maltose, and maltotriose) which are either used in food application or syrup industry. The industrial starch processing involves two high energy requiring steps: (1) Liquefaction (or gelatinization of starch molecules) which runs at very high temperature (105–110°C) for 5 min. (2) Saccharification (conversion of starch into simpler sugars) which requires the temperature at 55–60°C (Vieille and Zeikus, 2001). The raw starch binding thermostable amylases have become increasingly attractive to lower the process cost since they do not require gelatinized substrate for hydrolysis. The two thermophilic actinobacteria such as *Streptomyces* sp. (Kaneko et al., 2005) and *Streptomyces* sp. No. 4 (Primarini and Ohta, 2000), produce raw starch binding amylases which could be applied to reduce the energy input at industrial level making the overall process cost effective. Few other thermophilic actinobacteria are known to produce high maltotriose forming thermostable amylases which could be applicable in the food industries (listed in Table 5). Some alkaliphilic/alkalitolerant actinobacteria were reported to produce amylases functioning at alkaline pH, which are being used in detergent formulation to improve the detergency. At present, many modern laundries prefer amylase containing detergent for washing clothes at a lower temperature in order to save energy (Chakraborty et al., 2012).

#### Proteases

Proteases are one of the most important class of hydrolytic enzymes, which constitute >65% of the total industrial applications. A large array of actinobacterial species (including both alkalitolerant and alkaliphiles) produces alkalistable proteases and keratinase of commercial interest. The alkalistable proteases possess considerable applications in various industries such as detergent, leather, and food industries (Ellaiah et al., 2002). The alkalistable proteases are also used in the process of silver recovery from used X-ray or photographic film. The proteases of alkaliphilic actinobacteria are not only alkalistable but also thermostable (Gohel and Singh, 2012a), salt tolerant, and function actively in the presence of organic solvent (Thumar and Singh, 2009). The alkali-thermostable proteases could be a potent candidate in leather industries where the alkaline condition and high temperature are maintained during tanning process. In addition, salt and organic solvent tolerant proteases of actinobacteria find various applications in industrial processes requiring high salt concentration and solvents. The organic solvent tolerance increases the industrial value of proteases as organic solvents enhance the catalytic properties of hydrolytic enzymes (Klibanov, 2001) and preclude the occurrence of undesirable side reactions during the process.

#### Cellulases, xylanase, and acetyl xylan esterase

Cellulase and xylanase are the two industrially important enzymes that enable us to utilize the agricultural residues in generation of biofuel in a sustainable manner. The extreme operational conditions of industries demand highly thermostable enzymes. The two thermophilic actinobacteria, *Acidothermus cellulolyticus* (Mohagheghi et al., 1986) and *Thermobifida fusca* (Kim et al., 2005) are significantly fascinating the biofuel industry as well as several others (food, animal feed, textile, paper and pulp industry) as they are known to possess the robust enzymatic system to degrade cellulose and xylan fractions of lignocelluloic residues. The cellulases of *T. fusca* and *A. cellulyticus* have extensively been studied and are being used in bioethanol production from plant cell components. A cellulase from *T. fusca* has an additional advantage of extracting phenolics from apple peel, which can be used as antioxidants (Kim et al., 2005). This moderately thermophilic actinobacterium also secretes thermostable acetyl xylan esterase which catalyzes the removal of acetyl group from acetylxylan making easy access of xylanases to the substrate leading to its complete degradation (Yang and Liu, 2008). Thermostable and alkalistable enzymes capable of degrading lignocelluloic substrate have also been characterized from other thermophilic and alkaliphilic actinobacteria (listed in Table 5).

#### Dextranase

The process of sugar production from sugarcane juice requires high temperature and alkaline pH. The indigenous microorganisms present in the juice may produce dextran which needs to be degraded, otherwise it blocks the filter and slows down the clarification process, thus, decreasing the yield and quality of sugar produced (Purushe et al., 2012). Since the process occurs at high temperature and alkaline pH, the addition of alkalithermostable dextranase before processing can improve the yield as well as quality of sugar produced. Therefore, dextranase produced by some thermoalkaliphilic actinobacteria such as *Streptomyces* sp. NK458 is well-suited for such application (Purushe et al., 2012).

#### Nitrile hydratase

Another enzyme kown as nitrile hydratase has been reported from a large number of mesophilic and thermophilic actinobacteria, and is involved in the biotransformation of nitriles into useful compounds such as amines, amides, amidines, carboxylic acids, esters, aldehydes, and ketones (Banerjee et al., 2002). The industrial applicability of thermostable nitrile hydratases demands detailed investigation on enzymes from thermophilic actinobacteria. The thermostable nitrile hydratase from *Pseudonorcardia thermophila* has recently been immobilized in the gel matrix for acrylamide production (Martinez et al., 2014).

#### Laccase

Laccase catalyzes the oxidation of phenolics (2,6-dimethylphenylalanine and p-aminophenol) and produces colors, therefore, it is being used as a hair coloring agent. The coloring occurs best at alkaline pH, as in alkaline condition, hair tends to swell up leading to easy penetration of dye molecules. Therefore, an alkalistable laccase would be the best candidate to be used for such application. Actinobacteria are known to produce thermoalkalistable laccase (e.g., *Thermobifida fusca* BCRC 19214; Chen et al., 2013). Therefore, laccase can be produced from such actinobacterial strains for hair coloring application.

#### Alginate lyase

The alginate is a linear acidic polysaccharide and produced as a major component of cell wall of seaweeds. It consists of 1,4-linked α-d-mannuronate (M) and its epimer α-l-guluronate (G). These monomers polymerize in three ways: homopolymerization of G blocks [poly (G)] and homopolymerization of M blocks [poly (M)], and heteropolymerization of MG blocks [poly (MG)] (Gacesa, 1992). Alginate lyases act on these polymers to produce alginate oligosaccharides which can be used as therapeutic agents (anticoagulant, antitumor agent. and anti-inflammatory agent; Iwamoto et al., 2005). Alginate lyases are classified into two types (monofunctional and bifunctional) on the basis of their substrate specificity. Monofunctional enzymes can either act on poly(M) or poly(G) and bifunctional enzymes prefer the poly(MG) (Tondervik et al., 2010). But there are fewer reports on bifunctional and thermostable alginate lyase. An alkalitolerant actinobacterium, *Isoptericola halotolerans* CGMCC 5336 has been shown to produce moderately thermostable bifunctional alginate lyase (Dou et al., 2013).

#### Alditol oxidase

Oxidation of primary and secondary alcohols yields oxidative products that are used to synthesize other useful compounds. Chemical oxidation methods mediate the reaction by using heavy metals such as chromium and manganese. Interestingly, biocatalysts can also be employed to derive such oxidation reactions e.g., alcohol dehydrogenase. However, this enzyme requires NAD(P)^+^ as cofactor for the reaction which is very costly. To overcome this demerit, the research is being focused on isolating and characterizing thermostable flavoprotein alditol oxidase (AldO) from microbial sources for industrial applications. The gene of AldO of a thermophilic actinobacterium (*Acidothermus cellulolyticus*) was identified while searching for the homologs of the well-characterized AldO of *Streptomyces coelicolor* in the genome database (Winter et al., 2012). The gene of AldO was cloned and expressed in *E. coli* and the recombinant enzyme AldO displays a high thermostability (half-life at 75°C of 112 min) and requires cheaper molecular oxygen as terminal electron acceptor. Therefore, this enzyme can be used as an alternative of chemical catalysts in industrial processes.

#### Carbon monoxide dehydrogenase

Carbon monoxide dehydrogenase is an oxidoreductase enzyme that catalyzes the interconversion between carbon monoxide and carbon dioxide. This enzyme is produced in both anaerobic and aerobic microbes during autotrophic mode of nutrition. The enzyme has a great affinity to bind CO, thereby trapping the CO from the environment, therefore, can be applied in biofilters to purify these toxic gases released by industries. *Streptomyces* sp. G26 (Bell et al., 1988) and *Streptomyces thermoautotrophicus* (Gadkari et al., 1990) have been reported to produce the thermostable carbon monoxide dehydrogenase which is well-suited for filtering the hot air released from industries. This can also be employed in the biosensor to detect and quantitate atmospheric CO concentration.

#### Cutinase

Cutinase is a serine esterase that acts on the ester bonds of cutin (a component of cuticle layer of plant aerial parts). *Thermobifida fusca* produces two types of cutinases which display higher thermostability than the fungal cutinases (Chen et al., 2010). The enzyme exhibits broad substrate specificity such as plant cutin and soluble/insoluble esters and hydrolyzes them into hydroxyl and hydroxy epoxy fatty acids as end products. These fatty acids can be used as substrate in the enantioselective esterification reactions or in the production of phenolic compounds as well as the oil and dairy products. The enzyme can also metabolize the synthetic polyesters and other organic pollutants (Kleeberg et al., 2005), therefore, could be used in the *in vitro* biodegradation processes.

## Genome annotation, molecular insights, and genetic manipulation of thermophilic and alkaliphilic actinobacteria

The mechanisms, biosynthetic pathways and mode of action of several antibiotics of mesophilic origin have been elucidated. Classical random mutagenesis and rational genetic methods such as ribosome engineering, genome shuffling, down-regulation, and up-regulation of structural genes have been used to manipulate the genetic makeup of wild type actinobacteria strain for obtaining strains with desirable properties for e.g., enhancement in the antibiotic production titer (Olano et al., 2008). However, despite having prospective and novel characteristics, the biosynthetic pathways of bioactive compound and enzymatic system of the thermophilic and alkaliphilic actinobacteria are comparatively less explored. The inadequate information is available related to the heterologous gene expression, *in vitro* genetic engineering, structural elucidation and molecular insight on the catalysis of thermostable and alkalistable enzymes of actinobacteria. Only two thermophilic actinobacterial species, *Thermobifida fusca* and *Acidothermus cellulyticus* have been well-studied which are known to secrete a large array of highly thermostable and broad pH stable glycoside hydrolases. Their glycoside hydrolases are gaining considerable attention in the fuel biotechnology. The genes of thermo- or alkali-stable enzymes of some other thermophilic and alkaliphilic actinobacteria were cloned and expressed as well (shown in Table 6).

**Table 6:** **Summary of heterologous expression of proteins of thermophilic and alkaliphilic actinobacteria**.

**Actinobacteria**	**Enzymes**	**Expressi on Host**	**Optimum pH and temperature**	***K*_m_**	***V*_max_**	**References**
*Acidothermus cellulolyticus* 11B	Thermostable endoxylanase (Xyn10A)	*E. coli* BL21	pH 6 and 90°C	0.53 mg/ml	ND	Barabote et al., 2010
*Acidothermus cellulolyticus*	Endoglucanase (E1)	*Pichia pastoris*	pH 5.1 and 80°C	372 ± 50 μM	0.523 ± 0.070 μM/min	Lindenmuth and McDonald, 2011
*Acidothermus cellulolyticus*	Glucose isomerase	*E. coli* BL21	pH 6.5 and 80°C	0.40 M	6.41	Mu et al., 2012
*Acidothermus cellulolyticus*	Alditol oxidase	*E. coli* BL21	pH 6–9	Varies with different substrates	Varies with different substrates	Winter et al., 2012
*Saccharomonospora viridis*	Xylanase (Svixyn10A)	*E. coli* BL21	8.0 and 60°C	0.68 mg/ml	217.93 U/mg	Wang et al., 2012
*Nocardiopsis prasina* OPC-131	Chitinase	*E. coli* BL21	pH 9.0	ND	ND	Tsujibo et al., 2003
*Isoptericola jiangsuensis* CLG	Chitinases *IS-chiA* and *IS-chiB*	*E. coli* BL21	pH 5 and 30°C and pH 9 and 50°C, respectively	11.66 and 17 μM, respectively	10.93 and 12.24 μmol min^−1^ mg^−1^, respectively	Wu et al., 2011a
*Brachystreptospora xinjiangensis* OM-6 and *Nocardiopsis alba* OK-5	Proteases	*E. coli* BL21	pH 10	ND	ND	Gohel and Singh, 2012b
*Thermobifida fusca*	Cytochrome P450 monooxygenase CYP154H1	*E. coli* BL21	50°C	ND	ND	Schallmey et al., 2011
*Thermobifida fusca* TM51	β-D-mannosidase	*E. coli* BL21	7.17 and 53°C	180 μM	5.96 μmol min^−1^ mg^−1^,	Beki et al., 2003
*Thermobifida halotolerans*	Endoglucanase	*E. coli* BL21	pH 8 and 55°C	12.02 mg/ml	105.26 μM min^−1^	Zhang et al., 2011
*Thermobifida fusca*	Trehalose synthase	*Pichia pastoris*	25°C and pH 6.5	ND	ND	Wei et al., 2004
*Corynebacterium glutamicum*	Flavin containing monooxygenase	*E. coli* BL21	pH 8 and 25°C	Varies with substrate	Varies with substrate	Ameria et al., 2015

^*^ND, not determined.

The complete genome sequence analysis reveals the presence of genes encoding industrially useful enzymes or enzymes involved in the biosynthetic pathway of novel bioactive compounds (Velásquez and van der Donk, 2011). This also provides better understanding of the genetic makeup and cellular mechanisms of an organism which enables us to engineer microbes in order to enhance their efficacy for biotechnological purposes. The genome sequence of some important thermophilic and alkaliphilic actinobacteria were annotated and analyzed which provides some valuable information related to these microbes (summarized in Table 7). For instance, the genome annotation of cellulolytic actinobacterium, *Thermobifida fusca* revealed the presence of additional 29 putative glycoside hydrolases (cellulose-, dextran/starch-, and xylan-degrading enzymes) than the previously characterized glycosidases (Lykidis et al., 2007). This actinobacterium has been designated as a model organism for the cellulose degradation. *Thermobifida fusca* YX has been metabolically engineered to be used in biofuel production (Deng and Fong, 2011). The gene of bifunctional butyraldehyde/alcohol dehydrogenase (adhE2) from *Clostridium acetobutylicum* ATCC 824 was introduced into the genome of *T. fusca* to enhance its efficacy for cellulose degradation. This genetically engineered strain can utilize untreated lignocellulose and convert it directly into primary alcohols (1-propanol and 1-butanol). *T. fusca* is known to produce six structurally and functionally distinct cellulases (El–E6; Irwin et al., 1993). Out of these, the three enzymes [E1 (Cel9B), E2 (Cel6A), and E5 (Cel5A)] are β-(1, 4)-endoglucanases and catalyze the conversion of insoluble cellulose into cellobiose and other simpler sugars (Hu and Wilson, 1988). The other two cellulases such as E6 (Cel48A) and E3 (Cel6B) (Zhang et al., 1995) are β-(1,4)-exoglucanases and one cellulase E4 (Cel9A) has the ability to catalyze the endo- and exo-cellulysis. These six cellulases are produced in small quantities under uninduced conditions. But the constitutive expression of E2 was comparatively higher than others. The cellulase E2 has been shown to play a vital role in the early growth period of *T. fusca* (Spiridonov and Wilson, 1998). A transcriptional regulator CelR (340-residue polypeptide) binds to the operator (14-base pair inverted repeat) which is present in the upstream region of genes of six cellulases and represses the transcription of the cellulase genes in *T. fusca* (Spiridonov and Wilson, 1999). The binding of CelR is controlled by the presence of cellobiose which acts as an inducer and binds with repressor protein (CelR). Binding of cellobiose brings conformational changes in CelR protein and facilitates its dissociation from operators, thereby inducing the transcription of mRNA of cellulases. The cellulase Cel9A-90 (E4) shows highest activity among other cellulases in crystalline form. It has catalytic domain (CD) of a family 9 cellulases, a cellulose binding module (CBM3c), a fibronectin III-like domain, and a family 2 CBM domain (Li et al., 2010). A active site cleft is present in the CD that consists of six glucose binding sites, numbered from −4 to +2. These residues are aligned with a flat binding surface of the CBM3c. The mutein Cel9A-51 (without CBM3c) revealed the significant role of CBM3c in processivity of the enzyme. The enzymatic activity of Cel9A was shown to be enhanced upon replacement of a conserved residue (D513) of the CBM domain (Li et al., 2007b). A mutein Cel9A-68 was constructed by deleting CBM2 domain from a Cel9A-90 gene, which showed comparatively higher cellulolytic activity (Li et al., 2010). Another mutein Cel9A-68 (T245-L251) R252K (DEL) showed slightly improved filter paper activity and increased binding affinity toward bacterial microcrystalline cellulose (Zhou et al., 2004). An enzyme E5 (Cel5A) was found to be detergent stable, which has total six cysteine residues involved in the formation of three disulfide bonds. Among them, one bond is exposed outside which gets easily reduced to free sulfahydryl group while the other two bonds are not accessible. The reduction of one accessible bond does not affect the activity of an enzyme (McGinnis and Wilson, 1993). *Thermobifida fusca* also produces other thermostable enzymes (amylase, xylanase, and mannase). Xylanase reported from *T. fusca* is thermostable. Random mutagenesis was carried out to improve catalytic efficiency (12-fold increased), substrate affinity (4.5-fold decreased) and alkalistability of this xylanase. The thermostability of the mutein, however, decreased with the improvement of other functional characteristics (Wang and Xia, 2008).

**Table 7:** **General features of thermophilic and alkaliphilic actinobacteria genome**.

**Actinobacteria**	**Genome size (Mbp)**	**GC content (%)**	**Protein-coding genes**	**Genes involved in secondary metabolites metabolism**	**Genes involved in carbohydrate transport and metabolism**	**Unique characteristic**	**References**
*Thermobifida fusca* YX	~3.64	67.5	3117	–	–	It produces ~45 glycoside hydrolases	Lykidis et al., 2007
*Acidothermus cellulolyticus* 11B	~2.44	66.9	2157	–	–	It harbors a large array of industrial important enzymes	Barabote et al., 2009
*Thermobispora bispora* R51^T^	~4.2	72.43	3596	85	221	It contains two distinct transcriptionally active 16S rRNA genes	Liolios et al., 2010
*Thermomonospora curvata* B9^T^	~5.64	71.64	4985	181	161	It produces many bioactive compounds and thermostable enzymes	Chertkov et al., 2011
*Corynebacterium efficiens*	~3.15	63.4	2950	–	–	It produces industrially important amino acids	Nishio et al., 2003
*Rubrobacter radiotolerans* RSPS-4	~3.2 (circular genome and three plasmids)	66.91	3214	37	162	Extreme radiotolerant and moderately thermophilic actinobacterium	Egas et al., 2014
*Acidimicrobium ferrooxidans* ICP^T^	~2.16	68.29	2038	34	87	Iron reducing acidothermotolerant	Clum et al., 2009
*Saccharomonospora viridis* P101^T^	~4.31	67.32	3906	139	214	Causative agent of Farmer lung disease	Pati et al., 2009
*Saccharopolyspora rectivirgula*	~3.98	68.9	3840	–	–	Causative agent of Farmer lung disease	Pettersson et al., 2014
*Arthrobacter* sp. Strain IHBB 11108	3.6 (circular genome and plasmid)	58.97	3454	–	–	It produces alkalistable proteases	Kiran et al., 2015

Another cellulolytic actinobacterium, *Acidothermus cellulyticus*, is reported as a potent decomposer of plant cell material. The complete genome annotation revealed that it harbors 43 genes encoding carbohydrate active enzymes. Out of 43, total 35 proteins are glycoside hydrolases and remaining eight belong to carbohydrate esterases type. The 17 plant cell wall degrading enzymes (cellulolytic and hemicellulose hydrolysis), 10 fungal cell wall degrading enzymes (chitinases, N-acetylglucosaminidase, GH16 endo-1,3-beta-glucanase and others) and 16 other proteins (glycogen and trehalose synthesizing and degrading enzymes including GH13 family α-amylase) were identified from this actinobacterium. Among 43 enzymes, only 21 are actively secreted, while others are produced intracellularly (Barabote et al., 2009). The endoglucanase (E1 or Cel5A) of *A. cellulolyticus* is well-studied, which is ultra-thermostable, acid-stable, and displays higher substrate specificity (Tucker et al., 1989). The Cel5A belongs to glucoside hydrolases family 5 and 4/7 superfamily, and has been cloned in a number of hosts such as transgenic plants [tobacco (Dai et al., 2000), maize (Biswas et al., 2006), rice (Chou et al., 2011), and many others], and *Pichia pastoris* (Lindenmuth and McDonald, 2011). The endoglucanase producing transgenic plants ease the process of bioconversion of lignocellulosic materials into biofuels. The catalytic efficiency of Cel5A was increased by replacing of Tyr245 of WT-Cel5A with Gly (Y245G). This mutation reduces the end product inhibition and enhances the activity by 1480%. The mutein also releases 40% extra soluble sugar than wild type E1 enzyme (Baker et al., 2005). The gene of GH12 endoglucanase (not previously characterized) of *A. cellulolyticus* along with Cel5A gene were expressed into the *Zymomonas mobilis* to construct a consolidated bioprocessing (CBP) organism. Consolidated bioprocessing (CBP) is a new biotechnological approach to convert pretreated lignocellulosic materials to ethanol by using a single organism producing multiple hydrolytic enzymes (Linger et al., 2010).

Xylanase producing alkalitolerant actinobacterium, *Streptomyces viridochromogenes* strain M11 was isolated from marine sediment samples collected from the Xiaoping Island, China. This *Streptomyces* sp. produces thermostable and a broad pH stable xylanase. The xylanase production in this strain was increased (14% higher activity) by ribosome engineering. The ribosome engineering is an approach to introduce mutation in ribosome by using high concentrations of various antibiotics [10 times more concentration than minimal inhibitory concentration (MIC)]. This engineered strain produces antibiotic resistant mutants by causing mutation in the gene *rpsL* (ribosomal protein S12) and gene *rsmG* (16S rRNA methyltransferase). The K88R mutation of rpsL of this strain enhanced the xylanase production level (Liu et al., 2013). The UV mutants of *Streptomyces griseoaurantiacus* have also been shown to produce efficient cellulases (stable at high temperature and broad pH range) in relatively higher titers (Kumar, 2015). Crude oil degrading alkalitolerant actinobacterium, *Dietzia* strain DMYR9 has been isolated from oilfield and was metabolically engineered by irradiating with ^12^C^6+^ heavy ions to enhance its biodegradability (Zhou et al., 2013).

## Conclusions and future perspectives

Thermophilic and thermotolerant actinobacteria are found in 25 genera belonging to four major classes (*Actinobacteria, Acidimicrobiia, Rubrobacteria*, and *Thermoleophilia*). The taxonomic status of many thermophilic actinobacteria is ambiguous, therefore, has been revised repeatedly in the past. Bioprospecting of thermophilic actinobacteria represents an extensive pool of industrial and pharmaceutically relevant biomolecules. Their high abundance and metabolic versatility offer a new robust gateway to bioremediation of pollutants and organic residues. Although the first description on alkaliphilic actinobacteria appeared 70 years ago, the literature available on biodiversity, physiology, and ecology of alkaliphilic actinobacteria is quite inadequate. The growing industrial demand for alkalistable enzymes and biomolecules calls for further research on isolation, characterization, and bioprospecting of novel alkaliphilic actinobacteria. The use of metagenomic approaches will throw light on the novel genera of non-culturable actionobacteria and their genes in alkaline and hot environments. The availability of genome sequences of alkaliphilic and thermophilic actinobacteria is expected to encourage microbiologists and biotechnologists to go for gene mining that may lead to the discovery of novel biomolecules.

### Conflict of interest statement

The authors declare that the research was conducted in the absence of any commercial or financial relationships that could be construed as a potential conflict of interest.
